# Prophylaxis in Rhinitis Sympathetica*McKenzie—Transactions of the Medical and Chirurgical Faculty of the State of Maryland, 1885.

**Published:** 1886-12

**Authors:** S. O. Richey

**Affiliations:** Washington, D. C.


PROPHYLAXIS IN RHINITIS SYMPATHETICA.* *By* S. O. RICHEY, M.D.

In the evolution of medicine, gradual progress has been made to the present development of *preventive measures* in the management of disease, the practice of which, in its perfection, will be the science of the future. Prevention of disease, and the structural changes consequent, will, when we have the necessary knowledge, be more conservative, more simple, and more agreeable than our efforts to meet the disease in human bodies, disguised often by a complexity of symptoms, and to limit or prevent pathological metamorphoses.

Any rational being will prefer the proverbial preventive "ounce" rather than the "pound," later, and not upon the basis of quantity alone,—though even that might be deemed sufficient,—but upon that of the disagreeable environment and consequences of the disease.

There appear in medical journals from time to time elaborate discussions upon the most efficient means of relief of the *local* symptoms of hay-fever, or rhinitis sympathetica, by therapeutic measures or surgical procedures *after* structural changes have taken place in the nares; but very little is said in regard to prophylaxis, except the suggestion of change of climate—an offspring of the "pollen" dogma.

It is important that we get the idea of pollen out of our minds and accept the rational explanation, as will be done by all sooner or later, that *rhinitis sympathetica* is a reflex affection, or a peripheral expression of a more central disturbance, the impairment of the balance of circulation in the cervical plexus. Thus we have the influenza: the cause being slight,

*McKenzie—Transactions of the Medical and Chirurgical Faculty of the State of Maryland, 1885.

and acting interruptedly, the effect is a number of slight influenzas. The same cause acting persistently and gravely for a greater or less time produces what is called "a severe cold."

What is the cause of so distressing an affection? If it were pollen would it not be found among herbivora, and as much so during the winter season as in the summer among those domestic animals which are stabled and fed upon cured grass, from which the pollen is more likely to escape, and in greater abundance, than when it is green?

*Insufficient protection of the body, and especially the spinal region, from decided and quickly repeated alternations of temperature is the initial cause.*

In 1880–81 my attention was fixed by finding myself a sufferer from this affection. At that time the tone of my nervous system was very low, but not for the first time. I was also alternating between the city and the country, my days being spent in the city and my nights in the country, and in this I saw no sufficient cause for the disease, for others did the same without suffering, and I recovered with both these alleged causes persisting. Writers on the subject claim both as *predisposing* influences, the first of which I accept, and the latter I repudiate. With the dread of its direful consequences I naturally watched its course closely, in my own case, and studied the symptoms of the approaching crises until they became familiar to me. I looked up my books again for a hope of relief, but found it always resolved itself into seeking another climate for the season, and this my work would not allow. I found, too, that a change to the same place did not relieve all persons so affected, and that sometimes the same person would not be relieved at the same place, twice: These facts induced me to doubt whether any one would be safe from the supposed cause of the affection anywhere, until I met a young man who told me that he always found relief by spending the day in a boat on the lake, fishing, for there the *pollen* could not reach him. I then asked him if he wore the same clothes while fishing as at other times, and found that he replaced his *cotton-gauze undershirt* with a woolen shirt.

As I was then wearing the cotton-gauze undershirt, and remembering the slight creeping chills up my spine and at the nape of my neck prodromic of an attack, I put on a flannel undershirt, and with the exception of one or two attacks, immediately afterward, I have been well ever since.

The flannel being too warm for comfort in this climate during July, August and September, in looking through the furnishing establishments, I found an undershirt resembling in texture a fish-net, called in the shops "French netted goods," which I substituted with success and with perfect comfort. It does not, in its continuity, lie close to the body and rapidly absorb moisture, like the gauze; if it becomes damp, it does not dry out so rapidly, and thus by rapid and frequent evaporation, cause alternations of temperature and disturb the circulation of the spinal region with the reflex symptoms called rose-cold.

Those who change their abode in search of relief, go to a higher altitude or a more northern latitude, and while they do not thus escape "pollen," they find it comfortable and often necessary to wear more protective clothing next the skin, and in this fact, I believe, is to be found the cause of their relief. Any kind of material next the skin which will, like gauze, absorb sweat and dry out quickly, would produce the same consequences. One does not have this affection in the winter season, when well clad, unless from force of habit or an association of ideas in an impressionable individual. The affection is less frequent among women, because they perspire less and less frequently.

In the usual course of things my opportunities for observation of this affection are very limited as compared with those of others, being confined to those in whom it has caused *tubal catarrh*, whose impaired hearing could not be properly improved without removing the cause—the immediate cause being the rhinitis, producing congestion of the lining membrane by extension to the tube; the mediate cause being a disturbance of the spinal circulation. Laborers, especially in the country, in the midst of *pollen*, are less liable to have this trouble, for, being accustomed to constant healthful exercise, they have a more stable circulation, which is not so easily thrown out of balance.

While eight or ten cases are too limited a number to establish a theory, yet when taken with the fact that no one of them has failed in getting relief by *counsel* in the item of dress, they make this subject worthy of consideration.

It is not my object to discuss the treatment of symptoms, or the surgical management of nasal structural changes resulting from the persistence of this affection.

Let me ask, only, if it is best to *suppress* these peripheral manifestations by the use of cocaine, or the thermo-cautery? May not the affection, in such event, indicate its presence in some other and more serious way: in petit mal, for instance?

Is it not more exact and more scientific to find and remove such basal cause?

Washington, D. C.

